# Modern technologies and algorithms for scaffolding assembled genomes

**DOI:** 10.1371/journal.pcbi.1006994

**Published:** 2019-06-05

**Authors:** Jay Ghurye, Mihai Pop

**Affiliations:** Department of Computer Science and Center for Bioinformatics and Computational Biology, University of Maryland, College Park, Maryland, United States of America; University of Trento, ITALY

## Abstract

The computational reconstruction of genome sequences from shotgun sequencing data has been greatly simplified by the advent of sequencing technologies that generate long reads. In the case of relatively small genomes (e.g., bacterial or viral), complete genome sequences can frequently be reconstructed computationally without the need for further experiments. However, large and complex genomes, such as those of most animals and plants, continue to pose significant challenges. In such genomes, assembly software produces incomplete and fragmented reconstructions that require additional experimentally derived information and manual intervention in order to reconstruct individual chromosome arms. Recent technologies originally designed to capture chromatin structure have been shown to effectively complement sequencing data, leading to much more contiguous reconstructions of genomes than previously possible. Here, we survey these technologies and the algorithms used to assemble and analyze large eukaryotic genomes, placed within the historical context of genome scaffolding technologies that have been in existence since the dawn of the genomic era.

## Background

The increased availability and lower cost of DNA sequencing have revolutionized biomedical research. Thousands of humans have been sequenced to date, and genome sequencing is increasingly used in clinical practice, particularly in the context of cancer [1, 2]. Despite the long length of sequences generated by third-generation sequencing technologies (tens of thousands of base pairs), the automated reconstruction of entire genomes continues to be a formidable computational task, in no small part because of genomic repeats—ubiquitous features of eukaryotic genomes [3]. Recently, new genomic technologies have been developed that can "bridge" across repeats or other genomic regions that are difficult to sequence or assemble. We refer to technologies originally developed as a tool for interrogating the structure of genomes by cross-linking adjacent genomic segments and capturing these adjacencies through sequencing. These technologies are increasingly used to help improve genome assemblies by "scaffolding" together large segments of the genome. We survey here recent advances in this field, placed within the context of the technologies and algorithms that have been used for scaffolding throughout the entire genomic revolution. Note that our primary focus is on reconstructing the genome sequence of organisms rather than the structure of their chromosomes. Readers interested in the latter are referred to, e.g., [4].

**Fig 1:**
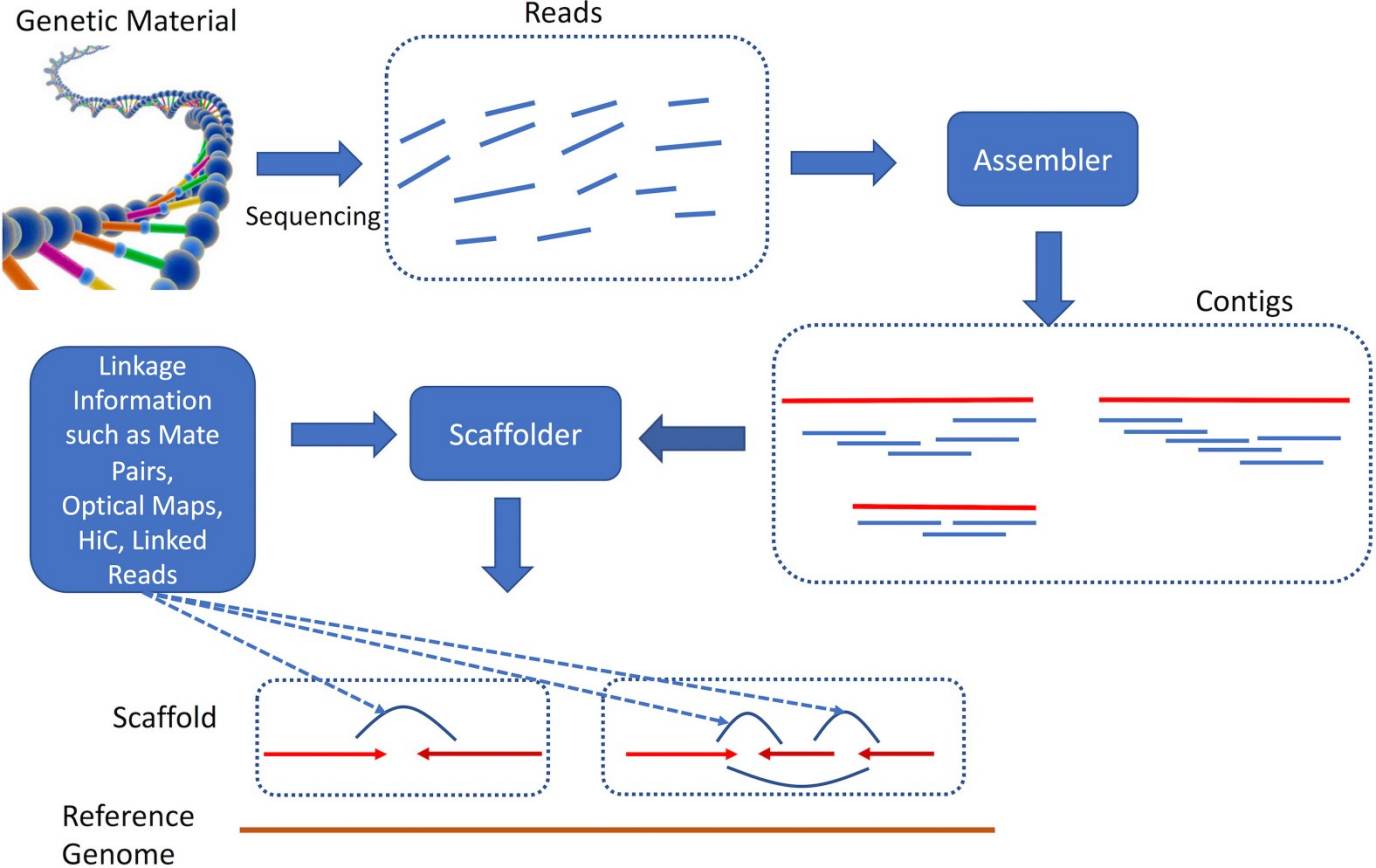
Overview of the genome assembly process. First, genetic material is sequenced, generating a collection of sequenced fragments (reads). These reads are processed by a computer program called an assembler, which merges the reads based on their overlap to construct larger contigs. Contigs are then oriented and ordered with respect to each other with a computer program called a scaffolder, relying on a variety of sources of linkage information. The scaffolds provide information about the long-range structure of the genome without specifying the actual DNA sequence within the gaps between contigs. The size of the gaps can also only be approximately estimated. contig, contiguous genomic segment.

Genome assembly (Fig 1)—the computational process used to reconstruct genomes from the relatively short DNA fragments that can be sequenced—is complicated mainly by genomic repeats [5–9]. These DNA segments that occur in two or more nearly identical copies within genomes induce ambiguity in the reconstruction of a genome, ambiguity that cannot be resolved with the information contained in the reads alone. Furthermore, genomes also contain regions with unusual base pair composition that are difficult to sequence. As a result, typical genome assemblies of eukaryotic genomes are highly fragmented, comprising tens to hundreds of thousands of contiguous genomic segments (contigs). This fact was recognized from the early days of genomics, and scientists have developed techniques that can generate information complementary to that contained in the reads. The assembly of the first living organism to be sequenced (*Haemophilus influenzae* [10]) relied on paired-read data that linked together relatively distant segments of the genome, allowing the assembled contigs to be ordered and oriented into a "scaffold" of the *H*. *influenzae* main chromosome [11–14].

Genome assembly approaches have been extensively reviewed [11–17], including recently [18]. Missing from this extensive body of literature is a focus on the algorithmic considerations underlying the use of long-range linking data in the assembly process. In this review, we highlight recent developments in the technologies used to generate long-range linking information and describe the computational algorithms that use this information to scaffold together the genomic segments generated by assembly algorithms. We place recent advances within the historical context of genome scaffolding technologies and algorithms and show how these technologies and algorithms have moved the field forward toward assembling larger and more complex eukaryotic genomes. We conclude with a survey of recent projects that demonstrate the effective combination of sequencing and scaffolding technologies to generate high-quality genome reconstructions.

## Sources of information for genome scaffolding

**Fig 2:**
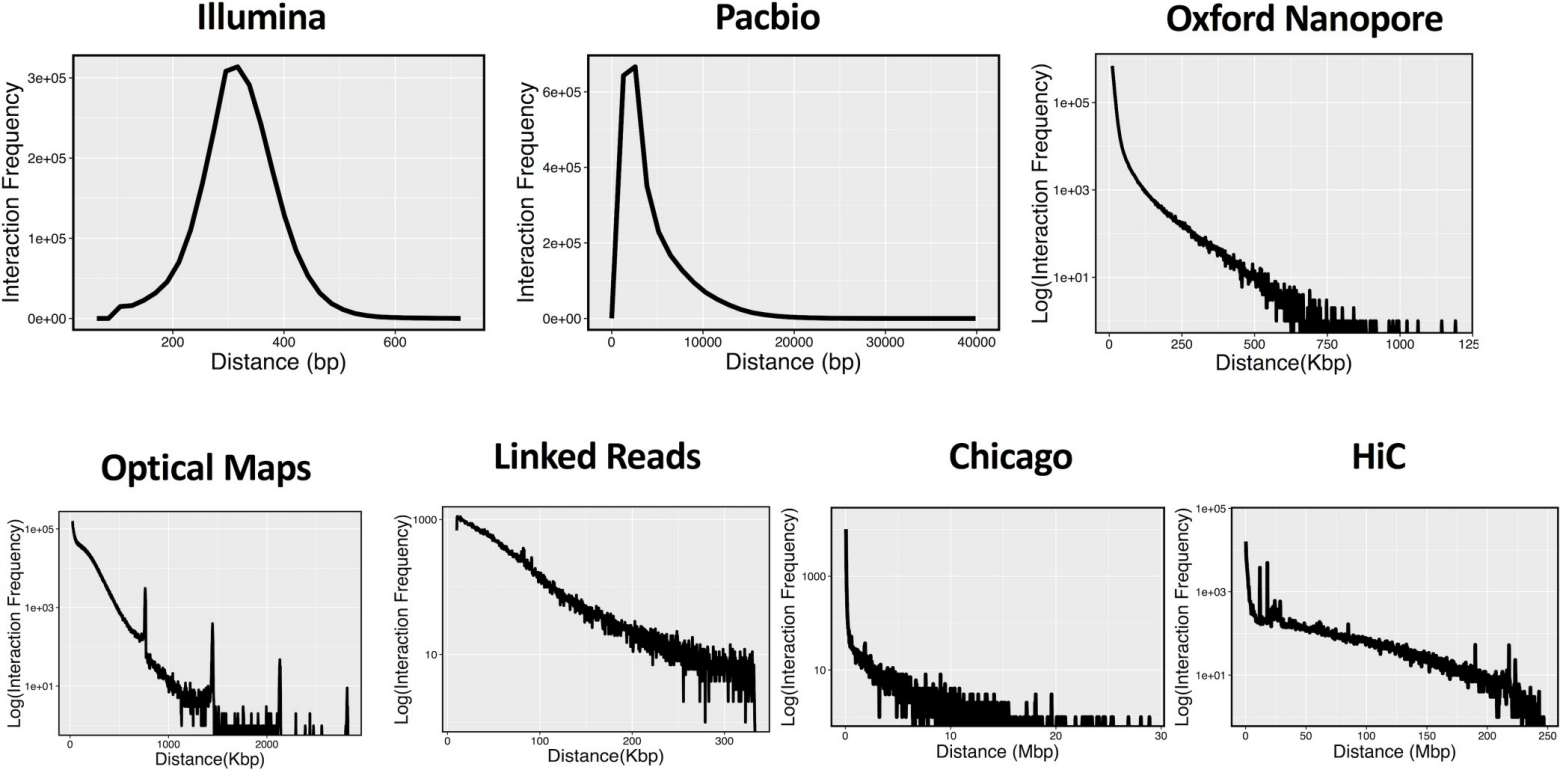
The genomic span covered by different technologies mentioned in this review. Reads and optical maps derived from the NA12878 sample (DNA from a human individual sequenced as part of the 1000 Genomes Project) were mapped to the GRCh38 human genome reference. The histograms represent as follows: Illumina—the separation between natively generated paired-end reads (SRX1049855); Pacbio—the length of the reads generated by the Pacific Biosciences technology (SRX1607993); Oxford Nanopore—the length of the reads generated by the Oxford Nanopore technology (https://github.com/nanopore-wgs-consortium/NA12878); optical maps—the length of the fragments mapped by the BioNano nanocoding technology (from BioNano website); linked reads—the span of the region covered by reads originating from the same DNA fragment, as generated by the 10X Genomics technology (SRX1392293); Chicago—the separation between read pairs generated by the Chicago chromosome conformation capture protocol (SRX1423027); and Hi-C—the separation between read pairs generated by the Hi-C chromosome conformation capture protocol (SRX3651893).

Broadly speaking, any type of information that hints at the relative location of genomic segments along a chromosome can be used to drive the scaffolding process. In most cases, the information used derives from genomic technologies specifically designed to interrogate the structure of chromosomes, though indirect inferences based on evolutionary arguments have also been used effectively in genome scaffolding. Fig 2 shows the extent to which different sequencing technologies can provide the linkage information for scaffolding. This linkage information can span anywhere from several hundreds to tens of thousands of base pairs (Illumina, Pacific Biosciences, and Oxford Nanopore) to hundreds of thousands of base pairs (linked reads and optical maps) to millions of base pairs (Chicago and Hi-C).

The genomic range spanned by the linking information is directly tied to the effectiveness of a particular technology to resolve certain classes of repeats [19]—to be effective, links must be longer than the length of a repeat but short enough so that they do not span multiple repeat units (which would increase the computational complexity of the scaffolding or repeat resolution process). Given the broad range of the lengths of repeats in most organisms, best results are usually obtained from a mixture of technologies or from technologies that yield data spanning a broad range of distances (such as third-generation sequencing reads, optical maps, or Hi-C links). Sequencing reads can be viewed as providing linking information that spans any distance within the length of the reads—as such, they provide valuable information for repeats of a broad range of sizes, up to the length of the reads.

We structure our presentation around the key types of information that can be used to organize genomic contigs into chromosome-wide scaffolds (Table 1).

**Table 1:** Comparison of different sequencing and mapping technologies.

Category	Scaffolding Data	Separation on the Genome	Orientation	Ordering	Distance
Physical mapping	Restriction maps	10–100 Kb	Yes	No	Yes
	Optical maps	10–100 Kb	Yes	Yes	Yes
Subcloning	10x Genomics	100 Kb	Yes	Yes	Yes
	Illumina TSLR	100 Kbp	Yes	Yes	Yes
Long-read data	Pacific Biosciences	10–15 Kb	Yes	Yes	Yes
	Oxford Nanopore	15–20 Kb	Yes	Yes	Yes
Paired read	Paired-end reads	100–500 bp	Yes	Yes	Yes
	Mate pairs	1,000–10,000 bp	Yes	Yes	Yes
Chromosome conformation	Hi-C	30–100 Mb	Yes	Yes	No
	Chicago	3–100 Mb	Yes	Yes	No
Synteny	Reference genome(s)	Up to genome size	Yes	Yes	Yes

Abbreviation: TSLR, TruSeq Synthetic Long Read.

### Physical mapping

Physical-mapping technologies attempt to estimate the location of specific loci along genomic chromosomes. The loci can be short DNA segments that are unique within the genome, as in the case of sequence-tagged sites (STSs)[20], or the recognition sequence of a restriction enzyme, as in the case of restriction mapping and optical mapping. The approximate location of the markers along chromosomes can be identified through a number of techniques, from fluorescence in situ hybridization (FISH)[21] to the analysis of the random breakage of DNA being exposed to X-rays (radiation hybrid mapping [22]) to direct measurement of restriction fragment sizes, as performed in restriction mapping [23]. The original use of restriction enzymes to map chromosomes resulted in unordered information—simply the list of sizes of the restriction fragments generated from the molecule—information that had to be converted into an ordered map through a complex computational process. Optical maps are an enhancement of restriction mapping, which provides the fragment order in addition to their size based on imaging the fluorescence of DNA molecules immobilized on a glass slide [24] or within a nanochannel [25] (the latter technology is called nanocoding).

**Fig 3:**
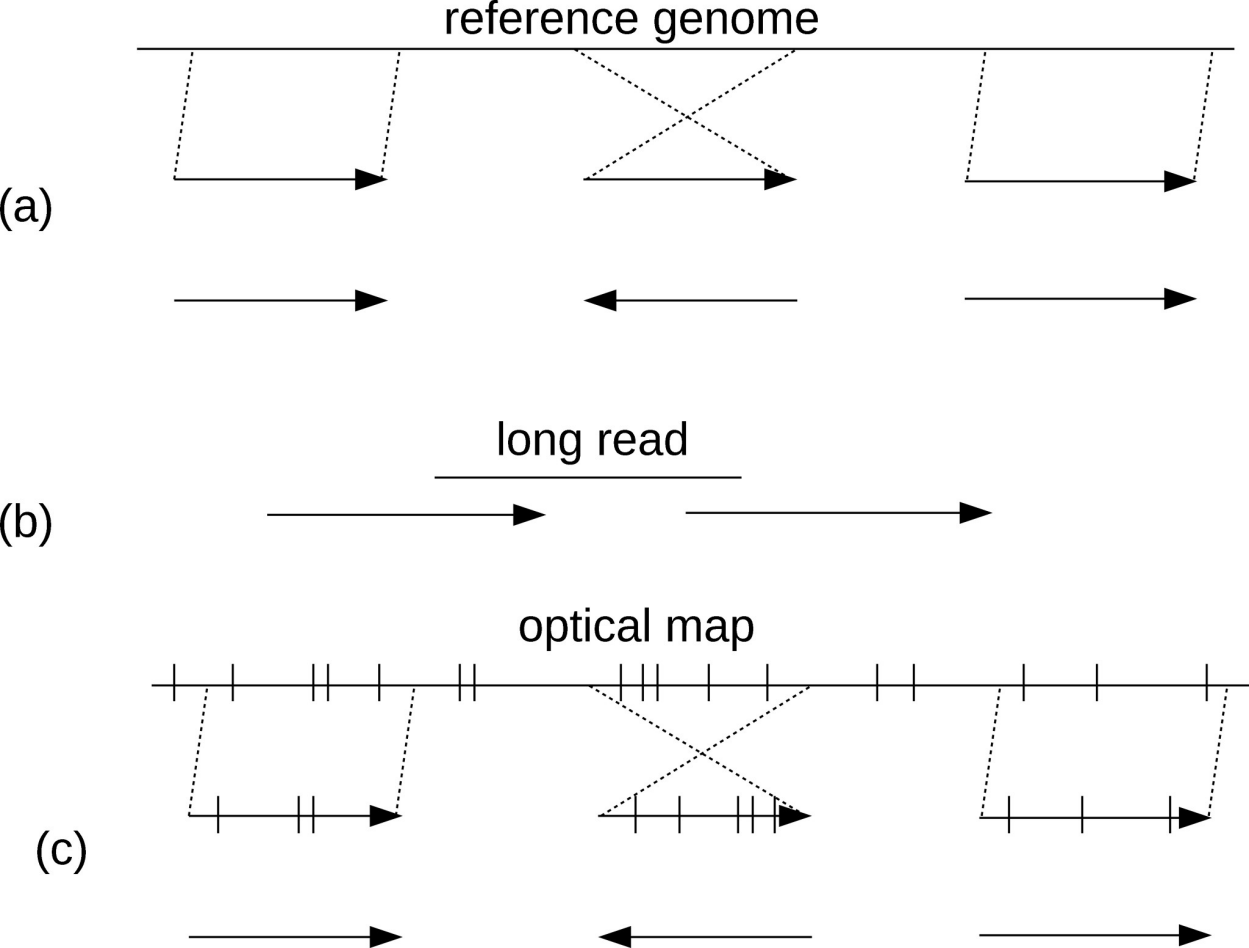
Mapping-based scaffolding approaches. (a) Contigs (arrows) are aligned to a reference genome, and their order and orientation is inferred from the alignment. (b) Long reads aligned to the ends of contigs imply their adjacency; (c) optical maps (tics represent location of restriction sites) can be used to infer the order and orientation of contigs (arrows) by aligning the inferred restriction pattern (tics within arrows) to that of the experimental map. contig, contiguous genomic segment.

Physical-mapping data is among the earliest technologies used to order genomic contigs along a chromosome [26–28]. The computational approach used to perform this task simply involves comparing experimentally derived maps to theoretical (in silico) maps generated from the sequenced contigs (Fig 3). This process is easiest when the landmarks being compared are distinguishable from each other (as is the case for STS and radiation hybrid maps) and substantially more complex and error prone for restriction maps, in which all the landmarks are identical in sequence, e.g., in the context of optical mapping [29].

The experimental maps themselves are often the result of assembling a collection of DNA fragments (clones) that have been mapped separately, leading to similar analytical challenges as those encountered in genome assembly [30]. In the case of unordered restriction maps, the assembly process is guided by the probability that two clones overlap, which is computed by taking into account the number of restriction fragments shared by the clones [31]. The pairwise overlap probabilities are used to assemble the clones into a chromosome-wide structure using a heuristic assembly algorithm (fingerprinted contigs [FPC]), which also allows for manual intervention to inspect and correct the resulting layout.

Ordered restriction maps, as generated by optical or nanocoding mapping, can be aligned using variants of dynamic programming alignment algorithms [32, 33]. In SOMA [34], fragment-sizing errors are penalized through a chi-squared scoring function, and a variant of a scheduling algorithm is used to determine the layout of contigs with ambiguous mappings. The runtime of the alignment algorithm used in SOMA scales with the fourth power of the number of restriction fragments, making the approach impractical for large genomes. Two recent approaches address this limitation. TWIN [35] relies on an extension of the FM-index [36] to speed up the alignment process, whereas Maligner [37] indexes the reference map by simulating the effect of common mapping errors such as false cuts or missed restriction sites.

### Subcloning

Subcloning involves breaking up the genome into large fragments that are then sequenced separately, retaining the connection between the sequencing reads generated from the same fragment (we refer to them as “linked reads” subsequently). The assembly process can then be run for each fragment separately, and the resulting assemblies can be merged together to reconstruct the full genome sequence. Initially, subcloning relied on bacterial artificial chromosomes (BACs) grown in *Escherichia coli*, yielding fragments in the range of ~100 kbp in length. The two ends of each BAC clone were sequenced first in order to construct a clone map [38] representing the relative relationship between individual BACs along the genome. From this information, a minimal tiling path was identified in order to decide which fragments would be fully sequenced. This strategy was used effectively in the early days of genomics, most notably during the public effort to sequence the human genome [39].

Recently, new technologies have been developed that perform the subcloning process in vitro. The technology from 10x Genomics partitions large DNA fragments into droplets, and the DNA is sheared, and sequencing libraries are constructed within the droplets. The DNA within each droplet is tagged with a droplet-specific barcode, and these barcoded DNA molecules then undergo sequencing, and a postprocessing algorithm parses the barcodes to group the reads originating from the same large DNA fragment [40]. The Illumina TruSeq Synthetic Long Read (TSLR) technology [41] is based on fragmenting DNA into large segments of about 10 kb in size, which are distributed into pools such that each pool contains a relatively small number of fragments (~200–300). Each pool is processed separately and barcoded with a unique barcode prior to sequencing.

When the original fragments have been sequenced deeply enough (which is usually the case for the TSLR technology and most applications of BACs), the pooled reads can be assembled together in order to create complete reconstructions of the individual fragments, effectively generating long and highly accurate sequencing reads. Several approaches have also been developed that rely on the unassembled pooled reads to guide the scaffolding process, approaches that can be effective even at low depths of sequencing coverage. Fragscaff [42], a method which was originally designed for contiguity-preserving transposition sequencing data, creates links between the ends of contigs, which are represented in the set of reads from the same pool. Within the resulting graph, Fragscaff then identifies a minimum spanning tree by using as edge weights the number of pools shared between contigs. The longest path within this tree is selected as the scaffold backbone. ARCS [43] maps the linked reads to the assembled contigs and constructs intercontig links by identifying pairs of contigs whose ends share sequences from the same read pool. Scaffolding is then performed with the LINKS scaffolder [44], a tool originally developed for scaffolding assemblies with the help of long-read data. A similar approach is used by ARKS [45], a tool that relies on k-mer matches instead of sequence alignment to infer the assignment of the linked reads to assembled contigs. In Supernova, Weisenfeld and colleagues [46] relied on pool-specific barcodes to construct an adjacency graph in which the nodes are the initial set of contigs or scaffolds and edges denote the number of pool-specific barcodes shared between scaffolds. In this graph, Supernova finds linear paths consistent with the links provided by barcodes and selects the highest-scoring path as the backbone of the final scaffold. Linked reads have also been used to identify errors in the assembly. Tigmint [47] flags as misassemblies regions of contigs where the depth of coverage by read pools (as inferred from the mapping of reads to assemblies) is lower than expected.

### Long-read data

Sequencing technologies that generate long sequencing reads, such as Pacific Bioscience [48] and Oxford Nanopore [49], can be seen as a special case of subcloning. Whereas genome assemblers such as CANU [50], FALCON [51], HINGE [52], MECAT [53], miniasm [54], and Flye [55] are effective in reconstructing genomic contigs from long-read data to achieve high-quality assemblies with only long-read data, the genome needs to be sequenced at considerably high coverage, incurring significant costs. A more cost-effective strategy involves supplementing a short-read assembly with a relatively low-coverage set of long-read data, effectively representing a collection of sparsely sampled genomic subclones. SSPACE-LongRead [56] was one of the earliest methods able to leverage long reads for scaffolding. This approach used BLASR [57] (an aligner tuned for the high error rates of third-generation sequencing technologies) to align contigs to the long reads in order to infer orientation, ordering, and the distance between contigs. SMSC [58] and BIGMAC [59] both start by using the long-read data to identify potential errors in the assembly, break the contigs at the boundaries of these errors, and then scaffold the resulting data using linking information inferred from the long reads. Because the alignment of long-read data is computationally intensive, LINKS [44] proposes an alignment-free approach that extracts pairs of k-mers separated by a predefined distance within long reads and then treats these as if they were paired short reads, relying on traditional scaffolding approaches. Unicycler [60] operates directly on the assembly graph generated by the SPAdes assembler [61] from short reads, using the long-read data to disambiguate paths through the graphs and generate longer, more accurate contigs. npScarf [62] leverages the real-time generation of data by nanopore sequencing devices to iteratively develop and improve the scaffolding of a genome as more data become available. The scaffolding algorithm operates in a greedy fashion, linking contigs together as soon as sufficient support is available in the set of reads and breaking prior links if new ones that contradict them have stronger support. This iterative greedy process can be stopped by the user once a sufficiently good assembly is generated, allowing the dynamic selection of the depth of sequencing depending on the actual quality of the resulting reconstruction.

### Paired-read technologies

By far, the most common source of information for scaffolding is technologies that yield information about the relative placement of pairs of reads along the genome being sequenced. Most commonly, this information is derived by carefully controlling DNA shearing prior to sequencing in order to obtain fragments of uniform sizes and by tracking the link between DNA sequences "read" from the same fragment. Multiple protocols have been developed to generate read-pairing information, and different names are commonly used to reflect the experimental source: paired-end reads (pairings natively generated by Illumina sequencing instruments, usually short range ~300–500 bp) and mate-pair or jumping libraries (pairing information derived with the help of additional experimental assays, usually spanning thousands to tens of thousands of base pairs). Here, we use these terms interchangeably, as the information being generated is the same—pairs of reads with an approximately known relative distance and orientation. The information provided by mate pairs can be used to link the contigs and produce scaffolds [63] or to guide the assembly process itself, allowing the effective resolution of repeats [19, 64].

**Fig 4:**
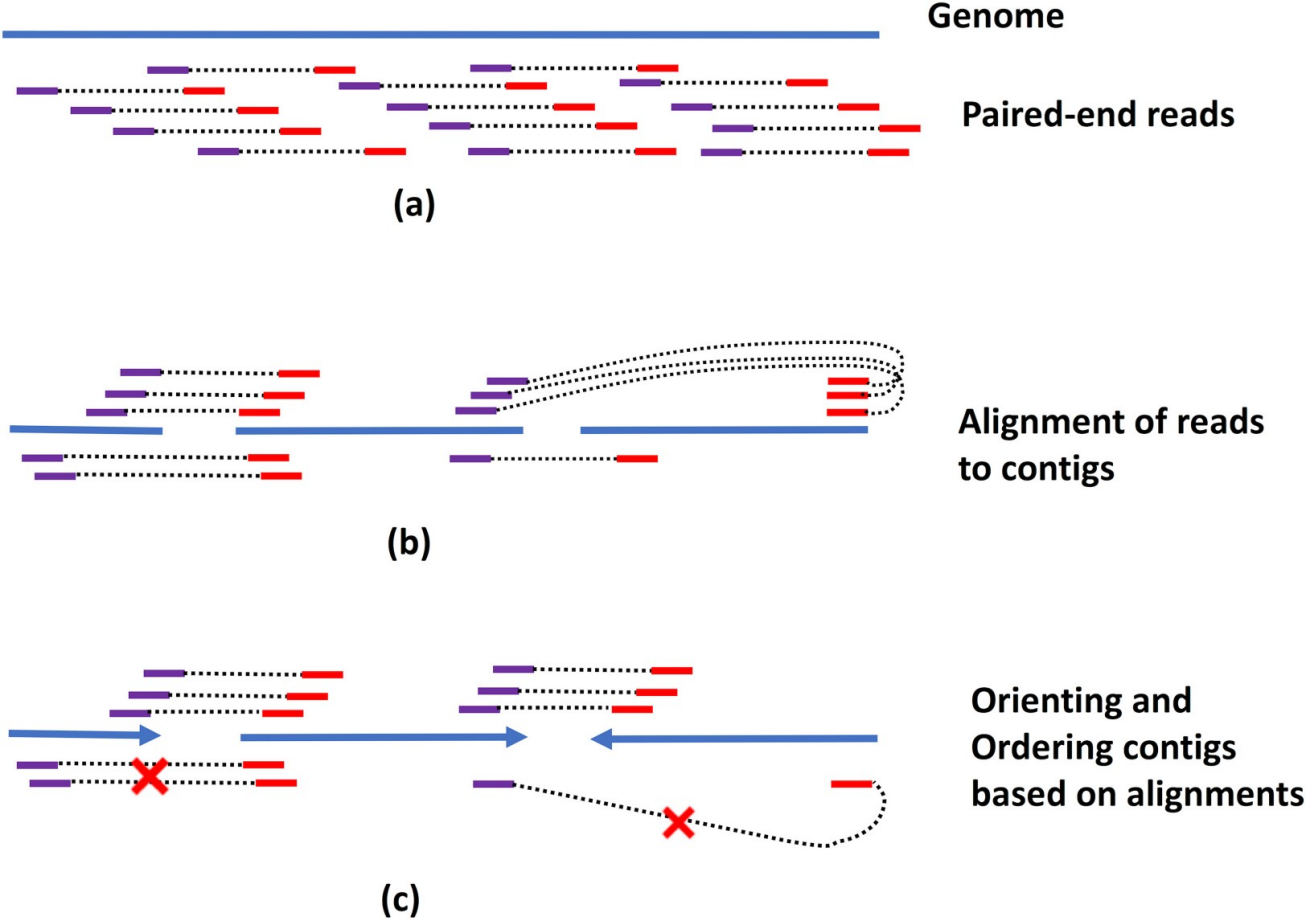
Use of pairwise linkage information for scaffolding. (a) Paired-end reads are sequenced from the genome. Depending on the technology, the approximate distance and/or relative orientation of the paired reads may not be known. (b) The reads are aligned to contigs. Reads with their ends aligned to two different contigs provide linkage information useful for scaffolding. (c) Linkage information is used to orient and order the contigs into scaffolds. Usually not all constraints can be preserved, and algorithms attempt to minimize inconsistencies (marked with X).

The algorithms for using mate-pair data in scaffolding genomes all follow a similar workflow. First, mate pairs whose ends map to different contigs are used to link together the corresponding contigs. Second, the pairwise linkage information is used to orient and order contigs with respect to each other. Third, the size of the gap between adjacent contigs is estimated from the experimentally determined size of the mate pairs, and a linear layout of the contigs along a scaffold is generated (Fig 4). Because contig orientation and ordering are computationally hard problems [7], scaffolders implement different greedy heuristics. Scaffolders such as MIP [65], SOPRA [66], and SCARPA [67] use integer programming to find the optimal orientation and ordering of contigs. Bambus [68] and SSPACE [69] use multiple libraries in a hierarchical manner to perform scaffolding, starting from libraries with smaller insert sizes (which are more accurate and yield a simpler problem) and progressively expanding scaffolds using libraries with larger insert sizes. OPERA-LG [70] uses a branch and bound search to determine the relative placement of contigs along the chromosome. The authors show that the size of the search space is bounded by the ratio between the library and contig size, implying that the branch and bound heuristic is efficient for the data typically encountered in practical applications despite a theoretically exponential complexity. The scaffolder inGAP-sf [71] merges information from both the assembly graph and paired-read data to construct scaffolds. In addition, this scaffolder introduces a statistical model for estimating the support for a link between two contigs, information that is used in constructing the scaffold. A number of tools have also been developed that use RNA sequencing (RNA-seq) data for scaffolding. Because of the long lengths of eukaryotic introns, such approaches can yield long-range genomic connections using standard short-length paired-end sequencing protocols, with the caveat that scaffolding is only effective in genic regions. Tools developed specifically for such data include RNAPATH [72], L_RNA [73], Rascaf [74], AGOUTI [75], and P_RNA [76].

In the context of repeat resolution, the orientation and distance constraints imposed by paired reads limit the number of possible traversals of the graph through a repeat region and can link together the unique genomic regions surrounding each instance of a repeat. Assemblers such as Velvet [77], ABySS [78], ABySS 2.0 [79], and IDBA-UD [80] use paired-end information to guide the walk through the assembly graph. SPAdes [81] and metaSPAdes [82] use the ratio of expected to observed numbers of mate pairs connecting two nodes [83] in the de Bruijn graph to check if the path traverses through a repetitive region. Wetzel and colleagues [19] explored the extent to which mate pairs can be used to resolve repetitive regions in prokaryotic genomes and showed that mate-pair libraries are most effective if tuned to the structure of the assembly graph.

### Chromosomal contact data

A special type of paired-read data is generated by techniques recently developed to study the three-dimensional structure of chromosomes inside a cell [84]. These techniques are collectively referred to as chromosomal conformation capture (C3), which generate pairwise linking information between reads that originate from genomic regions that are physically adjacent in a cell. Unlike mate-pair data, the distance and the relative orientation between the paired reads are not known a priori.

The two most commonly used protocols for capturing chromosome conformation are Hi-C [84] and Chicago [85]. In the Hi-C protocol, DNA in the cell nucleus is cross-linked and cut with a restriction enzyme. This process generates fragments of DNA that are distally located but physically associated with each other. The sticky ends of these fragments are biotinylated and then ligated to form a chimeric circle. The resulting circles are sheared and processed into sequencing libraries in which individual templates are chimeras of the physically associated DNA molecules. The Chicago protocol from Dovetail Genomics starts not with cells but with purified DNA so that any biologically associated interactions are eliminated. Artificial nucleosomes with random specificity are then used to condense the DNA into chromatin, which is then processed through the standard Hi-C protocol. The result is a collection of fragments that is enriched for sets of paired reads that capture long-range interactions between segments of DNA that were in contact within the artificial chromatin.

Because Hi-C and Chicago protocols do not provide estimates of the distance between the paired reads, the data can only be used to estimate the relative order and orientation of contigs and not the size of the gaps separating them. The scaffolding process starts by filtering the data to eliminate artifacts such as reads aligning to multiple locations or chimeric reads derived from the ligation junctions. Several tools have been developed for this purpose, including HiCUP [86], HiCPro [87], Juicer [88], Juicebox [89], and HiFive [90]. These tools align reads to the assembly using standard alignment programs [91, 92] and filter the alignments to remove experimental artifacts, yielding the “true” alignments, which imply the contact information. The number of paired reads linking two genomic regions (contact frequency) strongly correlates with the one-dimensional distance between the corresponding regions, thereby yielding an estimate of the relative placement of these segments within a genome. Furthermore, the contact frequency is much higher within a chromosome than across chromosomes, making it possible to infer chromosome structure directly from the genome assembly. Most of the algorithms developed to use Hi-C data for scaffolding use these properties to group contigs into chromosome-specific bins and then orient and order the contigs within each chromosome by maximizing the concordance with the experimentally derived contact frequencies. A major confounding factor in using Hi-C data for scaffolding is the nonrandom association between topological domains [93]. DNA-to-DNA interactions within the nucleus are organized in a domain structure in which interactions are much stronger within a domain than across domains. As a result, the Hi-C contact patterns exhibit a modular structure that can confound the estimate of distance between contigs during the scaffolding process.

DNATri [94] and LACHESIS [95] were the earliest methods developed to use Hi-C datasets for scaffolding. DNATri relies on a limited-memory Broyden–Fletcher–Goldfarb–Shanno optimization algorithm to identify the placement of contigs that best matches the contact frequencies derived from the Hi-C data. LACHESIS first clusters the contigs into chromosome groups using hierarchical clustering, matching a user-specified number of chromosomes. Then, it orders and orients contigs in each chromosome group/cluster separately by formulating the problem as identifying the "trunk" of a minimum spanning tree of the graph that encodes the Hi-C links between contigs. GRAAL [96] models the Hi-C data by distinguishing between *cis*- contacts (occurring within the same molecule) and *trans*- contacts (occurring across molecules). The contact frequency for the former are distance dependent, whereas the latter are drawn from a uniform probability distribution. The contigs are ordered and oriented to maximize the fit with this modeled data using a Metropolis optimization algorithm [97]. SALSA [98] relies on Hi-C data to correct misassemblies in the input contigs and then orients and orders the contigs using a maximal matching algorithm [98]. 3D-DNA [99] also corrects the errors in the input assembly and then iteratively orients and orders uniquely assembled contigs (unitigs) into a single megascaffold. This megascaffold is then broken into a user-specified number of chromosomes, identifying chromosomal ends based the on a Hi-C contact map. Putnam and colleagues [85] proposed a method called Hi-Rise that was specifically designed for handling Chicago libraries (based on artificial chromatin). They rely on a likelihood function that matches the characteristics of these data and use dynamic programming to identify a layout of contigs that maximizes the fit with the experimental data. Recently, Zhang and colleagues developed an approach for scaffolding polyploid genomes using Hi-C data in an approach called ALLHIC [100]. This approach relies upon the LACHESIS algorithm applied to Hi-C data that have been filtered to remove contacts that connect across haplotypes, thereby yielding haplotype-specific scaffolds.

## Practical considerations

The scaffolds generated with the help of the data described previously simply organize contigs along a genome without specifying the actual DNA sequence represented within the gap between adjacent contigs. Once the relative location of contigs is known, however, it is frequently easy to reconstruct the sequence within the gaps, a process that is commonly referred to as gap filling. Most commonly, mate-pair information is used to identify which reads could be placed within a gap, and then those reads are assembled to fill in the sequence within the gap, extending or even joining the adjacent contigs. Variants of this process are included in virtually all genome assemblers, e.g., ABySS [78], ALLPATHS-LG[101], and EULER [102], and several stand-alone solutions were also developed: GapFiller [103], SOAPdenovo GapCloser [104], and Sealer [105]. The latter approach relies on Bloom filters [106] to reduce memory usage, thereby enabling gap filling in large draft genomes. When long reads are available, gap filling can be performed with the help of reads that could not previously be incorporated in the assembly. The relatively higher quality of the contig sequences allows gap filling software to identify alignments that were missed during the assembly process. This principle is used by PBJelly [107] and GMCloser [108], approaches specifically developed for Pacific Biosciences data. GMCloser relies on a likelihood ratio test to determine the quality of alignments and remove poor-quality alignments that could lead to misassemblies.

Each of the data types used for scaffolding contain errors and have specific biases. Incorrect insert size estimates in mate-pair data can lead to ordering and gap estimation errors in scaffolds [109]. Hi-C data cannot provide accurate orientation information at small genomic distances, yielding small inversions within the scaffolds [110]. Optical mapping data have fairly low resolution and contain many errors, including incorrect estimates of fragment sizes and missed or spurious restriction cuts [111]. To reduce the impact of such errors on the ultimate quality of the assemblies, most studies rely on a combination of data sources. Long-read Pacific Biosciences data scaffolded with the help of nanocoding maps from BioNano Genomics were used by Pendleton and colleagues [112] to assemble a human genome and by Du and colleagues [113] to reconstruct the *indica* rice genome. Short-read data (Illumina) combined with 10x Genomics linked-read data and with optical maps were used by Mostoyov and colleagues [40] to generate a high-quality, haplotype-phased de novo assembly of a human genome. A combination of Pacific Biosciences, BioNano Genomics nanocoding maps, and Hi-C data were used to reconstruct the genome of the domestic goat [110]. Pacific Biosciences, BioNano Genomics nanocoding maps, and 10x Genomics linked-read data were effective in reconstructing a haplotype-phased version of the genome of a human individual [114]. A complex mixture of technologies, including BAC-based sequencing through short-read Illumina data, genetic map information, Hi-C data, and nanocoding maps, was used by Mascher and colleagues [115] to reconstruct the barley genome. Most recently, Jiao and colleagues [116] combined BioNano Genomics nanocoding maps, Hi-C data, and Pacific Biosciences long-read data to produce high-quality reconstructions of the genomes of three relatives of *Arabidopsis thaliana*. Recently, Moll and colleagues [117] used the model legume *Medicago trunculata* as a basis for critically evaluating the impact of different technologies and their combinations on the quality of the resulting genome reconstruction. Their analysis revealed the effectiveness of Dovetail Chicago data followed by the use of BioNano Genomics nanocoding mapping information in improving the original assembly generated from Pacific Biosciences reads.

## Leveraging synteny—A missed opportunity

Synteny refers to the colocalization of genes or genomic loci along a chromosome. In many cases, whereas the DNA sequence itself may diverge significantly during evolution, related organisms often preserve synteny and gene order. The conservation of synteny can thus be used to help order contigs along a chromosome by inferring their placement based on the location within a related genome of the orthologs of the genes found in the contigs. Despite the rapid increase of complete and draft genomes in public databases, the use of synteny information in genome reconstruction has not been widely adopted. Here, we provide a brief overview of the approaches that have been developed in this field in hopes of spurring increased use of these data in genome projects.

Synteny-based methods first map contigs onto the reference genomes using a whole-genome aligner [118, 119]. The orientation and ordering of contigs are then inferred from the alignment data (Fig 3). Methods such as OSLay [120], ABACAS [121], Mauve Aligner [122], fillScaffolds [123], r2cat [124], and CAR [125] use only one reference genome for scaffolding draft assemblies. They are primarily based on mapping the assembly to a complete or incomplete reference genome and attempt to identify the ordering and orientation of contigs that are most consistent with the reference genome.

The main challenges in using synteny data to guide genome reconstruction involve reconciling true differences with the reference genome as well as handling incomplete reference genomes. Such approaches may lead to mistakes if the reference genome is rearranged with respect to the genome being assembled or if the two genomes are too distant in phylogenetic terms. To address such challenges, MeDuSa [126] and Ragout [127] use multiple reference genomes along with the phylogenetic tree of these genomes as a reference to scaffold contigs. MeDuSa models the problem of using multiple reference genomes for scaffolding as an instance of maximum weight path cover problem [128], which is known to be NP-hard (computational problem for which no known polynomial time algorithms exist), and proposes a greedy heuristic to find an approximate solution. Ragout [129] and Ragout 2 [130] represent the target and reference genomes as a multicolored breakpoint graph with nodes representing the conserved synteny blocks and edges representing the adjacency of these blocks. In this graph, Ragout finds the missing adjacencies by solving a half-breakpoint state parsimony problem on the given phylogenetic tree and then orients and orders synteny blocks to reconstruct the target genome. Multi-CAR [131] starts by processing each reference genome separately using CAR. Multi-CAR then reconciles the different contig orderings by constructing a graph in which nodes are contigs and edges are the adjacencies given by different reference genomes. A maximal weight perfect matching [132] within this graph defines the final set of scaffolds.

## Perspective

As we have shown, technological advances on both the experimental and computational sides have dramatically improved the ability of reconstructing the genomes of complex eukaryotic organisms, including repeat-rich plants such as rice and barley. Most of the technologies used today are evolved versions of approaches developed decades ago during the dawn of the genomic era. Although read lengths have increased, the accuracy of optical and nanocoding maps has improved, and subcloning approaches are now performed in vitro without the need for culturing the DNA in an *E*. *coli* host; the fundamental properties of the data being generated have not changed in a meaningful way. The exceptions are the technologies used to interrogate the structure of chromosomes through sequencing—Hi-C and related approaches. The paired reads generated by these technologies no longer provide per-pair distance constraints; rather, distance information can only be reconstructed from the frequency of "contacts" between distant sections of the genome being reconstructed. In return, however, these technologies provide much longer-range linking information than provided by any other technology. Just as long-read technologies have dramatically advanced our ability to reconstruct genomes, the long-range linking information provided by Hi-C and similar technologies has made it possible to link together complete chromosome arms [99, 110].

In the near future, it is likely that many previously intractable genomes will be reconstructed with the help of long-read sequencing data coupled with paired-read information from chromosome conformation capture technologies, augmented by short-read and short mate-pair technologies aimed at resolving the small-scale structure of genomes. This opportunity is particularly relevant to scientists studying the complex genomes of plants [133] or insects [134] for which few genomic resources are currently available.

As we have already mentioned, a largely unused source of information is the sequences of the many genomes already sequenced and deposited in public databases. This vast source of data can be a valuable addition to the other types of genomic data being used in genome reconstruction, particularly in projects aiming to more densely sample particular regions of the tree of life (e.g., the genomes of cereal crops [133, 135]).

In our review, we have omitted a discussion of experimental challenges or cost, in part because our primary focus has been on algorithmic considerations and in part because of the rapid changes in technologies that would make any cost estimates obsolete even before the ink has dried on the paper. In general, such practical aspects are insufficiently discussed in current literature, and the community would benefit from a review focused on the technical challenges and costs of the available technologies.

Generating haplotype-phased chromosome-scale assemblies of eukaryotic genomes with the mix of sequencing technologies has been the driving force behind the development of the newer genome assembly methods. Koren and colleagues [136] proposed a method called “trio-binning” that uses short and accurate Illumina reads from two parental genomes to partition long and noisy reads from an offspring into a haplotype-specific set of reads, and each haplotype is then assembled independently.

It is conceivable that in the very near future, further developments in genomic technologies will make the automatic reconstruction of mammalian genomes possible. Recent advances in nanopore sequencing devices are already yielding longer reads than all prior technologies, potentially leading to the ability to assemble complete eukaryotic genomes from nanopore data alone. Rather than the end of a road, such developments will create the opportunity for scientists to tackle even harder challenges, such as the complete reconstruction of individual haplotypes, particularly in the context of heterogeneous mixtures such as tumors or microbial mixtures or polyploid genomes. Some progress is being made in haplotyping human genomes with the help of pedigree information (specifically trios comprising two parents and a child) [136]; however, the solution to the more complex problems posed by mixtures and polyploidy will require further developments in both genomic technologies, such as those outlined in our review, as well as in the design of algorithms and tools able to effectively leverage the information provided by these technologies.

## References

[1] Alexandrov LB, Nik-Zainal S, Wedge DC, Aparicio SAJR, Behjati S, Biankin AV, et al Signatures of mutational processes in human cancer. Nature. 2013;500(7463):415–21. 10.1038/nature12477 PubMed Central PMCID: PMCPMC3776390. 23945592 PMC3776390

[2] Kasar S, Kim J, Improgo R, Tiao G, Polak P, Haradhvala N, et al Whole-genome sequencing reveals activation-induced cytidine deaminase signatures during indolent chronic lymphocytic leukaemia evolution. Nat Commun. 2015;6:8866 10.1038/ncomms9866 PubMed Central PMCID: PMCPMC4686820. 26638776 PMC4686820

[3] Alkan C, Sajjadian S, Eichler EE. Limitations of next-generation genome sequence assembly. Nature Methods. 2010;8:61 10.1038/nmeth.1527 21102452 PMC3115693

[4] Barutcu AR, Fritz AJ, Zaidi SK, van Wijnen AJ, Lian JB, Stein JL, et al C-ing the Genome: A Compendium of Chromosome Conformation Capture Methods to Study Higher-Order Chromatin Organization. 2016;231(1):31–5. 10.1002/jcp.25062 26059817 PMC4586368

[5] Medvedev P, Georgiou K, Myers G, Brudno M. Computability of Models for Sequence Assembly In: Giancarlo R, Hannenhalli S, editors. Algorithms in Bioinformatics. WABI 2007. Lecture Notes in Computer Science, vol 4645 Berlin: Springer; 2007 p. 289–301.

[6] Tarhio J, Ukkonen E. A greedy algorithm for constructing shortest common superstrings In: Gruska J, Rovan B, Wiedermann J, editors. Mathematical Foundations of Computer Science 1986. MFCS 1986. Lecture Notes in Computer Science, vol 233 Berlin: Springer; 1986 p. 602–10.

[7] Kececioglu JD, Myers EW. Combinatorial algorithms for DNA sequence assembly. Algorithmica. 1995;13(1–2):7–51. 10.1007/bf01188580

[8] Schmid M, Frei D, Patrignani A, Schlapbach R, Frey JE, Remus-Emsermann MNP, et al Pushing the limits of de novo genome assembly for complex prokaryotic genomes harboring very long, near identical repeats. Nucleic Acids Research. 2018;46(17):8953–65. 10.1093/nar/gky726 30137508 PMC6158609

[9] Jain M, Koren S, Miga KH, Quick J, Rand AC, Sasani TA, et al Nanopore sequencing and assembly of a human genome with ultra-long reads. Nature Biotechnology. 2018;36:338 10.1038/nbt.4060 29431738 PMC5889714

[10] Fleischmann RD, Adams MD, White O, Clayton RA, Kirkness EF, Kerlavage AR, et al Whole-genome random sequencing and assembly of Haemophilus influenzae Rd. Science. 1995;269(5223):496–512. . 7542800 10.1126/science.7542800

[11] Nagarajan N, Pop M. Sequence assembly demystified. Nat Rev Genet. 2013;14(3):157–67. 10.1038/nrg3367 23358380

[12] Ghurye JS, Cepeda-Espinoza V, Pop M. Metagenomic Assembly: Overview, Challenges and Applications. Yale J Biol Med. 2016;89(3):353–62. PubMed Central PMCID: PMCPMC5045144. 27698619 PMC5045144

[13] Miller JR, Koren S, Sutton G. Assembly algorithms for next-generation sequencing data. Genomics. 2010;95(6):315–27. 10.1016/j.ygeno.2010.03.001 PubMed Central PMCID: PMCPMC2874646. 20211242 PMC2874646

[14] Simpson JT, Pop M. The Theory and Practice of Genome Sequence Assembly. Annu Rev Genomics Hum Genet. 2015;16:153–72. 10.1146/annurev-genom-090314-050032 25939056

[15] Schatz MC, Delcher AL, Salzberg SL. Assembly of large genomes using second-generation sequencing. 2010;20(9):1165–73. 10.1101/gr.101360.109 20508146 PMC2928494

[16] Alkan C, Coe BP, Eichler EE. Genome structural variation discovery and genotyping. Nature Reviews Genetics. 2011;12:363 10.1038/nrg2958 21358748 PMC4108431

[17] Chaisson MJP, Wilson RK, Eichler EE. Genetic variation and the de novo assembly of human genomes. Nature Reviews Genetics. 2015;16:627 10.1038/nrg3933 26442640 PMC4745987

[18] Sedlazeck FJ, Lee H, Darby CA, Schatz MC. Piercing the dark matter: bioinformatics of long-range sequencing and mapping. Nature Reviews Genetics. 2018;19(6):329–46. 10.1038/s41576-018-0003-4 29599501

[19] Wetzel J, Kingsford C, Pop M. Assessing the benefits of using mate-pairs to resolve repeats in de novo short-read prokaryotic assemblies. BMC Bioinformatics. 2011;12:95 10.1186/1471-2105-12-95 PubMed Central PMCID: PMCPMC3103447. 21486487 PMC3103447

[20] Williams BD, Schrank B, Huynh C, Shownkeen R, Waterston RH. A genetic mapping system in Caenorhabditis elegans based on polymorphic sequence-tagged sites. Genetics. 1992;131(3):609–24. PubMed Central PMCID: PMCPMC1205034. 1321065 10.1093/genetics/131.3.609 PMC1205034

[21] Wu R, Shi Z-R. Comparison of Chromogenic in situ Hybridization, Fluorescence in situ Hybridization, and Immunohistochemistry. Handbook of Immunohistochemistry and in Situ Hybridization of Human Carcinomas. Cambridge, MA: Elsevier Academic Press; 2002 p. 13–26.

[22] Lawrence S, Morton NE, Cox DR. Radiation hybrid mapping. Proceedings of the National Academy of Sciences. 1991;88(17):7477–80. 10.1073/pnas.88.17.7477 1881887 PMC52323

[23] Schwartz DC, Li X, Hernandez LI, Ramnarain SP, Huff EJ, Wang YK. Ordered restriction maps of Saccharomyces cerevisiae chromosomes constructed by optical mapping. Science. 1993;262(5130):110–4. . 8211116 10.1126/science.8211116

[24] Cai W, Aburatani H, Stanton VP Jr., Housman DE, Wang YK, Schwartz DC. Ordered restriction endonuclease maps of yeast artificial chromosomes created by optical mapping on surfaces. Proc Natl Acad Sci U S A. 1995;92(11):5164–8. 10.1073/pnas.92.11.5164 7761468 PMC41869

[25] Lam ET, Hastie A, Lin C, Ehrlich D, Das SK, Austin MD, et al Genome mapping on nanochannel arrays for structural variation analysis and sequence assembly. Nat Biotechnol. 2012;30(8):771–6. 10.1038/nbt.2303 PubMed Central PMCID: PMCPMC3817024. 22797562 PMC3817024

[26] Fickett JW, Cinkosky MJ. A Genetic Algorithm for Assembling Chromosome Physical Maps. Bioinformatics, Supercomputing and Complex Genome Analysis Proceedings of the Second International Conference on Bioinformatics, Supercomputing, and Complex Genome Analysis. St. Petersburg, FL: World Scientific; 1993 p. 273–85.

[27] Gillett W, Daues J, Hanks L, Capra R. Fragment collapsing and splitting while assembling high-resolution restriction maps. J Comput Biol. 1995;2(2):185–205. 10.1089/cmb.1995.2.185 7497126

[28] Kohara Y, Akiyama K, Isono K. The physical map of the whole E. coli chromosome: application of a new strategy for rapid analysis and sorting of a large genomic library. Cell. 1987;50(3):495–508. 3038334 10.1016/0092-8674(87)90503-4

[29] Engler FW, Hatfield J, Nelson W, Soderlund CA. Locating sequence on FPC maps and selecting a minimal tiling path. Genome Res. 2003;13(9):2152–63. 10.1101/gr.1068603 PubMed Central PMCID: PMCPMC403717. 12915486 PMC403717

[30] Golumbic MC, Kaplan H, Shamir R. On the Complexity of DNA Physical Mapping. Adv Appl Math. 1994;15(3):251–61. 10.1006/aama.1994.1009

[31] Soderlund C, Longden I, Mott R. FPC: a system for building contigs from restriction fingerprinted clones. Comput Appl Biosci. 1997;13(5):523–35. 9367125 10.1093/bioinformatics/13.5.523

[32] Anantharaman TS, Mishra B, Schwartz DC. Genomics via Optical Mapping II: Ordered Restriction Maps. J Comput Biol. 1997;4(2):91–118. 10.1089/cmb.1997.4.91 9228610

[33] Valouev A, Schwartz DC, Zhou S, Waterman MS. An algorithm for assembly of ordered restriction maps from single DNA molecules. Proc Natl Acad Sci U S A. 2006;103(43):15770–5. 10.1073/pnas.0604040103 PubMed Central PMCID: PMCPMC1635078. 17043225 PMC1635078

[34] Nagarajan N, Read TD, Pop M. Scaffolding and validation of bacterial genome assemblies using optical restriction maps. Bioinformatics. 2008;24(10):1229–35. 10.1093/bioinformatics/btn102 PubMed Central PMCID: PMCPMC2373919. 18356192 PMC2373919

[35] Muggli M, Puglisi S, Boucher C. Efficient Indexed Alignment of Contigs to Optical Maps In: Brown D, Morgenstern B, editors. Algorithms in Bioinformatics. Lecture Notes in Computer Science. Berlin: Springer; 2014 p. 68–81.

[36] Ferragina P, Manzini G. Opportunistic data structures with applications. Proceedings 41st Annual Symposium on Foundations of Computer Science; 2000 Nov 12–14; Redondo Beach, CA. Piscataway, NJ: IEEE; 2000.

[37] Mendelowitz LM, Schwartz DC, Pop M. Maligner: a fast ordered restriction map aligner. Bioinformatics. 2016;32(7):1016–22. 10.1093/bioinformatics/btv711 PubMed Central PMCID: PMCPMC4907389. 26637292 PMC4907389

[38] Rowen L, Mahairas G, Hood L. Sequencing the human genome. Science. 1997;278(5338):605–7. Epub 1998/02/12. . 9381170 10.1126/science.278.5338.605

[39] Lander ES, Linton LM, Birren B, Nusbaum C, Zody MC, Baldwin J, et al Initial sequencing and analysis of the human genome. Nature. 2001;409(6822):860–921. 10.1038/35057062 . 11237011

[40] Mostovoy Y, Levy-Sakin M, Lam J, Lam ET, Hastie AR, Marks P, et al A hybrid approach for de novo human genome sequence assembly and phasing. Nat Methods. 2016;13(7):587–90. 10.1038/nmeth.3865 PubMed Central PMCID: PMCPMC4927370. 27159086 PMC4927370

[41] Voskoboynik A, Neff NF, Sahoo D, Newman AM, Pushkarev D, Koh W, et al The genome sequence of the colonial chordate, Botryllus schlosseri. eLife. 2013;2:e00569 Epub 2013/07/11. 10.7554/eLife.00569 23840927 PMC3699833

[42] Adey A, Kitzman JO, Burton JN, Daza R, Kumar A, Christiansen L, et al In vitro, long-range sequence information for de novo genome assembly via transposase contiguity. Genome Res. 2014;24(12):2041–9. 10.1101/gr.178319.114 PubMed Central PMCID: PMCPMC4248320. 25327137 PMC4248320

[43] Yeo S, Coombe L, Warren RL, Chu J, Birol I. ARCS: scaffolding genome drafts with linked reads. Bioinformatics. 2018;34(5):725–31. 10.1093/bioinformatics/btx675 29069293 PMC6030987

[44] Warren RL, Yang C, Vandervalk BP, Behsaz B, Lagman A, Jones SJM, et al LINKS: Scalable, alignment-free scaffolding of draft genomes with long reads. 2015;4(1):35 10.1186/s13742-015-0076-3 26244089 PMC4524009

[45] Coombe L, Zhang J, Vandervalk BP, Chu J, Jackman SD, Birol I, et al ARKS: chromosome-scale scaffolding of human genome drafts with linked read kmers. 2018;19(1):234 10.1186/s12859-018-2243-x 29925315 PMC6011487

[46] Weisenfeld NI, Kumar V, Shah P, Church DM, Jaffe DB. Direct determination of diploid genome sequences. Genome Res. 2017;27(5):757–67. 10.1101/gr.214874.116 28381613 PMC5411770

[47] Jackman SD, Coombe L, Chu J, Warren RL, Vandervalk BP, Yeo S, et al Tigmint: correcting assembly errors using linked reads from large molecules. 2018;19(1):393 10.1186/s12859-018-2425-6 30367597 PMC6204047

[48] Levene MJ, Korlach J, Turner SW, Foquet M, Craighead HG, Webb WW. Zero-mode waveguides for single-molecule analysis at high concentrations. Science. 2003;299(5607):682–6. 10.1126/science.1079700 12560545

[49] Jain M, Olsen HE, Paten B, Akeson M. The Oxford Nanopore MinION: delivery of nanopore sequencing to the genomics community. Genome Biol. 2016;17(1):239 10.1186/s13059-016-1103-0 PubMed Central PMCID: PMCPMC5124260. 27887629 PMC5124260

[50] Koren S, Walenz BP, Berlin K, Miller JR, Bergman NH, Phillippy AM. Canu: scalable and accurate long-read assembly via adaptive k-mer weighting and repeat separation. 2017 10.1101/gr.215087.116 28298431 PMC5411767

[51] Chin C-S, Peluso P, Sedlazeck FJ, Nattestad M, Concepcion GT, Clum A, et al Phased diploid genome assembly with single-molecule real-time sequencing. Nature Methods. 2016;13:1050 10.1038/nmeth.4035 27749838 PMC5503144

[52] Kamath GM, Shomorony I, Xia F, Courtade T, Tse DN. HINGE: Long-read assembly achieves optimal repeat resolution. 2017 10.1101/gr.216465.116 28320918 PMC5411769

[53] Xiao C-L, Chen Y, Xie S-Q, Chen K-N, Wang Y, Han Y, et al MECAT: fast mapping, error correction, and de novo assembly for single-molecule sequencing reads. Nature Methods. 2017;14:1072 10.1038/nmeth.4432 28945707

[54] Li H. Minimap and miniasm: fast mapping and de novo assembly for noisy long sequences. Bioinformatics. 2016;32(14):2103–10. Epub 2016/05/07. 10.1093/bioinformatics/btw152 PubMed Central PMCID: PMCPMC4937194. 27153593 PMC4937194

[55] Kolmogorov M, Yuan J, Lin Y, Pevzner P. Assembly of Long Error-Prone Reads Using Repeat Graphs. BioRxiv [Preprint]. 2018 Available from: 10.1101/247148. 30936562

[56] Boetzer M, Pirovano W. SSPACE-LongRead: scaffolding bacterial draft genomes using long read sequence information. BMC Bioinformatics. 2014;15:211 10.1186/1471-2105-15-211 PubMed Central PMCID: PMCPMC4076250. 24950923 PMC4076250

[57] Chaisson MJ, Tesler G. Mapping single molecule sequencing reads using basic local alignment with successive refinement (BLASR): application and theory. BMC Bioinformatics. 2012;13:238 10.1186/1471-2105-13-238 PubMed Central PMCID: PMCPMC3572422. 22988817 PMC3572422

[58] Zhu S, Chen DZ, Emrich SJ. Single molecule sequencing-guided scaffolding and correction of draft assemblies. BMC Genomics. 2017;18(Suppl 10):879 Epub 2017/12/16. 10.1186/s12864-017-4271-8 29244003 PMC5731603

[59] Lam K-K, Hall R, Clum A, Rao SJBB. BIGMAC: breaking inaccurate genomes and merging assembled contigs for long read metagenomic assembly. 2016;17(1):435 10.1186/s12859-016-1288-y 27793084 PMC5084376

[60] Wick RR, Judd LM, Gorrie CL, Holt KE. Unicycler: Resolving bacterial genome assemblies from short and long sequencing reads. PLoS Comput Biol.2017;13(6):e1005595 10.1371/journal.pcbi.1005595 28594827 PMC5481147

[61] Bankevich A, Nurk S, Antipov D, Gurevich AA, Dvorkin M, Kulikov AS, et al SPAdes: A New Genome Assembly Algorithm and Its Applications to Single-Cell Sequencing. J Comput Biol. 2012;19(5):455–77. Epub 2012/04/18. 10.1089/cmb.2012.0021 22506599 PMC3342519

[62] Cao MD, Nguyen SH, Ganesamoorthy D, Elliott AG, Cooper MA, Coin LJ. Scaffolding and completing genome assemblies in real-time with nanopore sequencing. Nature communications. 2017;8:14515 Epub 2017/02/22. 10.1038/ncomms14515 28218240 PMC5321748

[63] Huson DH, Reinert K, Myers EW. The greedy path-merging algorithm for contig scaffolding. J ACM. 2002;49(5):603–15. 10.1145/585265.585267

[64] Ghurye J, Pop M. Better Identification of Repeats in Metagenomic Scaffolding In: Frith M, Storm Pedersen C, editors. Algorithms in Bioinformatics. Lecture Notes in Computer Science. Berlin: Springer; 2016 p. 174–84.

[65] Salmela L, Makinen V, Valimaki N, Ylinen J, Ukkonen E. Fast scaffolding with small independent mixed integer programs. Bioinformatics. 2011;27(23):3259–65. 10.1093/bioinformatics/btr562 21998153 PMC3223363

[66] Dayarian A, Michael TP, Sengupta AM. SOPRA: Scaffolding algorithm for paired reads via statistical optimization. BMC Bioinformatics. 2010;11:345 10.1186/1471-2105-11-345 PubMed Central PMCID: PMCPMC2909219. 20576136 PMC2909219

[67] Donmez N, Brudno M. SCARPA: scaffolding reads with practical algorithms. Bioinformatics. 2013;29(4):428–34. 10.1093/bioinformatics/bts716 23274213

[68] Pop M, Kosack DS, Salzberg SL. Hierarchical scaffolding with Bambus. Genome Res. 2004;14(1):149–59. 10.1101/gr.1536204 PubMed Central PMCID: PMCPMC314292. 14707177 PMC314292

[69] Boetzer M, Henkel CV, Jansen HJ, Butler D, Pirovano W. Scaffolding pre-assembled contigs using SSPACE. Bioinformatics. 2011;27(4):578–9. 10.1093/bioinformatics/btq683 21149342

[70] Gao S, Bertrand D, Chia BKH, Nagarajan N. OPERA-LG: efficient and exact scaffolding of large, repeat-rich eukaryotic genomes with performance guarantees. Genome Biol. 2016;17:102 10.1186/s13059-016-0951-y PubMed Central PMCID: PMCPMC4864936. 27169502 PMC4864936

[71] Shi W, Ji P, Zhao F. The combination of direct and paired link graphs can boost repetitive genome assembly. Nucleic Acids Research. 2017;45(6):e43–e. 10.1093/nar/gkw1191 27924003 PMC5399794

[72] Mortazavi A, Schwarz EM, Williams B, Schaeffer L, Antoshechkin I, Wold BJ, et al Scaffolding a Caenorhabditis nematode genome with RNA-seq. Genome Res. 2010;20(12):1740–7. Epub 2010/10/29. 10.1101/gr.111021.110 20980554 PMC2990000

[73] Xue W, Li JT, Zhu YP, Hou GY, Kong XF, Kuang YY, et al L_RNA_scaffolder: scaffolding genomes with transcripts. BMC Genomics. 2013;14:604 Epub 2013/09/10. 10.1186/1471-2164-14-604 24010822 PMC3846640

[74] Song L, Shankar DS, Florea L. Rascaf: Improving Genome Assembly with RNA Sequencing Data. Plant Genome. 2016;9(3). Epub 2016/12/03. 10.3835/plantgenome2016.03.0027 . 27902792

[75] Zhang SV, Zhuo L, Hahn MW. AGOUTI: improving genome assembly and annotation using transcriptome data. GigaScience. 2016;5(1):31 Epub 2016/07/21. 10.1186/s13742-016-0136-3 27435057 PMC4952227

[76] Zhu BH, Xiao J, Xue W, Xu GC, Sun MY, Li JT. P_RNA_scaffolder: a fast and accurate genome scaffolder using paired-end RNA-sequencing reads. BMC Genomics. 2018;19(1):175 Epub 2018/03/04. 10.1186/s12864-018-4567-3 29499650 PMC5834899

[77] Zerbino DR, McEwen GK, Margulies EH, Birney E. Pebble and rock band: heuristic resolution of repeats and scaffolding in the velvet short-read de novo assembler. PLoS ONE. 2009;4(12):e8407 10.1371/journal.pone.0008407 PubMed Central PMCID: PMCPMC2793427. 20027311 PMC2793427

[78] Simpson JT, Wong K, Jackman SD, Schein JE, Jones SJ, Birol I. ABySS: A parallel assembler for short read sequence data. Genome Res. 2009;19(6):1117–23. 10.1101/gr.089532.108 19251739 PMC2694472

[79] Jackman SD, Vandervalk BP, Mohamadi H, Chu J, Yeo S, Hammond SA, et al ABySS 2.0: Resource-efficient assembly of large genomes using a Bloom filter. Genome Res. 2017 10.1101/gr.214346.116 28232478 PMC5411771

[80] Peng Y, Leung HCM, Yiu SM, Chin FYL. IDBA-UD: a de novo assembler for single-cell and metagenomic sequencing data with highly uneven depth. Bioinformatics. 2012;28(11):1420–8. 10.1093/bioinformatics/bts174 22495754

[81] Bankevich A, Nurk S, Antipov D, Gurevich AA, Dvorkin M, Kulikov AS, et al SPAdes: a new genome assembly algorithm and its applications to single-cell sequencing. J Comput Biol. 2012;19(5):455–77. 10.1089/cmb.2012.0021 PubMed Central PMCID: PMCPMC3342519. 22506599 PMC3342519

[82] Nurk S, Meleshko D, Korobeynikov A, Pevzner PA. metaSPAdes: a new versatile metagenomic assembler. Genome Res. 2017;27(5):824–34. 10.1101/gr.213959.116 28298430 PMC5411777

[83] Prjibelski AD, Vasilinetc I, Bankevich A, Gurevich A, Krivosheeva T, Nurk S, et al ExSPAnder: a universal repeat resolver for DNA fragment assembly. Bioinformatics. 2014;30(12):i293–301. 10.1093/bioinformatics/btu266 PubMed Central PMCID: PMCPMC4058921. 24931996 PMC4058921

[84] Lieberman-Aiden E, van Berkum NL, Williams L, Imakaev M, Ragoczy T, Telling A, et al Comprehensive mapping of long-range interactions reveals folding principles of the human genome. Science. 2009;326(5950):289–93. 10.1126/science.1181369 PubMed Central PMCID: PMCPMC2858594. 19815776 PMC2858594

[85] Putnam NH, O'Connell BL, Stites JC, Rice BJ, Blanchette M, Calef R, et al Chromosome-scale shotgun assembly using an in vitro method for long-range linkage. Genome Res. 2016;26(3):342–50. 10.1101/gr.193474.115 26848124 PMC4772016

[86] Wingett S, Ewels P, Furlan-Magaril M, Nagano T, Schoenfelder S, Fraser P, et al HiCUP: pipeline for mapping and processing Hi-C data. F1000Res. 2015 10.12688/f1000research.7334.1 26835000 PMC4706059

[87] Servant N, Varoquaux N, Lajoie BR, Viara E, Chen C-J, Vert J-P, et al HiC-Pro: an optimized and flexible pipeline for Hi-C data processing. Genome Biol. 2015;16:259 10.1186/s13059-015-0831-x PubMed Central PMCID: PMCPMC4665391. 26619908 PMC4665391

[88] Durand NC, Shamim MS, Machol I, Rao SSP, Huntley MH, Lander ES, et al Juicer Provides a One-Click System for Analyzing Loop-Resolution Hi-C Experiments. Cell Syst. 2016;3(1):95–8. 10.1016/j.cels.2016.07.002 27467249 PMC5846465

[89] Durand NC, Robinson JT, Shamim MS, Machol I, Mesirov JP, Lander ES, et al Juicebox Provides a Visualization System for Hi-C Contact Maps with Unlimited Zoom. Cell Syst. 2016;3(1):99–101. Epub 2016/07/29. 10.1016/j.cels.2015.07.012 27467250 PMC5596920

[90] Sauria MEG, Phillips-Cremins JE, Corces VG, Taylor J. HiFive: a tool suite for easy and efficient HiC and 5C data analysis. Genome Biol. 2015;16:237 10.1186/s13059-015-0806-y PubMed Central PMCID: PMCPMC5410870. 26498826 PMC5410870

[91] Langmead B, Salzberg SL. Fast gapped-read alignment with Bowtie 2. Nat Methods. 2012;9(4):357–9. 10.1038/nmeth.1923 22388286 PMC3322381

[92] Li H, Durbin R. Fast and accurate short read alignment with Burrows-Wheeler transform. Bioinformatics. 2009;25(14):1754–60. 10.1093/bioinformatics/btp324 PubMed Central PMCID: PMCPMC2705234. 19451168 PMC2705234

[93] Dixon JR, Selvaraj S, Yue F, Kim A, Li Y, Shen Y, et al Topological domains in mammalian genomes identified by analysis of chromatin interactions. Nature. 2012;485:376 10.1038/nature11082 22495300 PMC3356448

[94] Kaplan N, Dekker J. High-throughput genome scaffolding from in vivo DNA interaction frequency. Nat Biotechnol. 2013;31(12):1143–7. 10.1038/nbt.2768 PubMed Central PMCID: PMCPMC3880131. 24270850 PMC3880131

[95] Burton JN, Adey A, Patwardhan RP, Qiu R, Kitzman JO, Shendure J. Chromosome-scale scaffolding of de novo genome assemblies based on chromatin interactions. Nat Biotechnol. 2013;31(12):1119–25. 10.1038/nbt.2727 PubMed Central PMCID: PMCPMC4117202. 24185095 PMC4117202

[96] Marie-Nelly H, Marbouty M, Cournac A, Flot J-F, Liti G, Parodi DP, et al High-quality genome (re)assembly using chromosomal contact data. Nat Commun. 2014;5:5695 10.1038/ncomms6695 PubMed Central PMCID: PMCPMC4284522. 25517223 PMC4284522

[97] Metropolis N. Monte Carlo: In the beginning and some great expectations In: Alcouffe R, Dautray R, Forster A, Ledanois G, Mercier B, editors. Monte-Carlo Methods and Applications in Neutronics, Photonics and Statistical Physics. Lecture Notes in Physics, vol 240 Springer: Berlin; 1985 p. 62–70.

[98] Ghurye J, Pop M, Koren S, Bickhart D, Chin C-S. Scaffolding of long read assemblies using long range contact information. BMC Genomics. 2017;18(1):527 10.1186/s12864-017-3879-z 28701198 PMC5508778

[99] Dudchenko O, Batra SS, Omer AD, Nyquist SK, Hoeger M, Durand NC, et al De novo assembly of the genome using Hi-C yields chromosome-length scaffolds. Science. 2017;356(6333):92–5. 10.1126/science.aal3327 PubMed Central PMCID: PMCPMC5635820. 28336562 PMC5635820

[100] Zhang J, Zhang X, Tang H, Zhang Q, Hua X, Ma X, et al Allele-defined genome of the autopolyploid sugarcane Saccharum spontaneum L. Nature Genetics. 2018;50(11):1565–73. 10.1038/s41588-018-0237-2 30297971

[101] Gnerre S, Maccallum I, Przybylski D, Ribeiro FJ, Burton JN, Walker BJ, et al High-quality draft assemblies of mammalian genomes from massively parallel sequence data. Proc Natl Acad Sci U S A. 2011;108(4):1513–8. 10.1073/pnas.1017351108 PubMed Central PMCID: PMCPMC3029755. 21187386 PMC3029755

[102] Pevzner PA, Tang H, Waterman MS. An Eulerian path approach to DNA fragment assembly. Proc Natl Acad Sci U S A. 2001;98(17):9748–53. 10.1073/pnas.171285098 PubMed Central PMCID: PMCPMC55524. 11504945 PMC55524

[103] Boetzer M, Pirovano W. Toward almost closed genomes with GapFiller. Genome Biol. 2012;13(6):R56 10.1186/gb-2012-13-6-r56 PubMed Central PMCID: PMCPMC3446322. 22731987 PMC3446322

[104] Luo R, Liu B, Xie Y, Li Z, Huang W, Yuan J, et al SOAPdenovo2: an empirically improved memory-efficient short-read de novo assembler. Gigascience. 2012;1(1):18 10.1186/2047-217X-1-18 PubMed Central PMCID: PMCPMC3626529. 23587118 PMC3626529

[105] Paulino D, Warren RL, Vandervalk BP, Raymond A, Jackman SD, Birol I. Sealer: a scalable gap-closing application for finishing draft genomes. BMC Bioinformatics. 2015;16:230 10.1186/s12859-015-0663-4 PubMed Central PMCID: PMCPMC4515008. 26209068 PMC4515008

[106] Bloom BH. Space/time trade-offs in hash coding with allowable errors. Commun ACM. 1970;13(7):422–6. 10.1145/362686.362692

[107] English AC, Richards S, Han Y, Wang M, Vee V, Qu J, et al Mind the gap: upgrading genomes with Pacific Biosciences RS long-read sequencing technology. PLoS ONE. 2012;7(11):e47768 10.1371/journal.pone.0047768 PubMed Central PMCID: PMCPMC3504050. 23185243 PMC3504050

[108] Kosugi S, Hirakawa H, Tabata S. GMcloser: closing gaps in assemblies accurately with a likelihood-based selection of contig or long-read alignments. Bioinformatics. 2015;31(23):3733–41. 10.1093/bioinformatics/btv465 26261222

[109] Murphy RR, O'Connell J, Cox AJ, Schulz-Trieglaff O. NxRepair: error correction in de novo sequence assembly using Nextera mate pairs. PeerJ. 2015;3:e996 10.7717/peerj.996 PubMed Central PMCID: PMCPMC4458127. 26056623 PMC4458127

[110] Bickhart DM, Rosen BD, Koren S, Sayre BL, Hastie AR, Chan S, et al Single-molecule sequencing and chromatin conformation capture enable de novo reference assembly of the domestic goat genome. Nat Genet. 2017;49(4):643–50. 10.1038/ng.3802 28263316 PMC5909822

[111] Mendelowitz L, Pop M. Computational methods for optical mapping. GigaScience. 2014;3(1):33 10.1186/2047-217X-3-33 25671093 PMC4323141

[112] Pendleton M, Sebra R, Pang AWC, Ummat A, Franzen O, Rausch T, et al Assembly and diploid architecture of an individual human genome via single-molecule technologies. Nat Methods. 2015;12(8):780–6. 10.1038/nmeth.3454 PubMed Central PMCID: PMCPMC4646949. 26121404 PMC4646949

[113] Du H, Yu Y, Ma Y, Gao Q, Cao Y, Chen Z, et al Sequencing and de novo assembly of a near complete indica rice genome. Nat Commun. 2017;8:15324 10.1038/ncomms15324 28469237 PMC5418594

[114] Seo J-S, Rhie A, Kim J, Lee S, Sohn M-H, Kim C-U, et al De novo assembly and phasing of a Korean human genome. Nature. 2016;538(7624):243–7. 10.1038/nature20098 27706134

[115] Mascher M, Gundlach H, Himmelbach A, Beier S, Twardziok SO, Wicker T, et al A chromosome conformation capture ordered sequence of the barley genome. Nature. 2017;544(7651):427–33. 10.1038/nature22043 28447635

[116] Jiao W-B, Accinelli GG, Hartwig B, Kiefer C, Baker D, Severing E, et al Improving and correcting the contiguity of long-read genome assemblies of three plant species using optical mapping and chromosome conformation capture data. Genome Res. 2017;27(5):778–86. 10.1101/gr.213652.116 28159771 PMC5411772

[117] Moll KM, Zhou P, Ramaraj T, Fajardo D, Devitt NP, Sadowsky MJ, et al Strategies for optimizing BioNano and Dovetail explored through a second reference quality assembly for the legume model, Medicago truncatula. BMC genomics. 2017;18(1):578 10.1186/s12864-017-3971-4 . 28778149 PMC5545040

[118] Delcher AL, Salzberg SL, Phillippy AM. Using MUMmer to identify similar regions in large sequence sets. Curr Protoc Bioinformatics. 2003;Chapter 10:Unit 10.3. 10.1002/0471250953.bi1003s00 18428693

[119] Rahmani A-M, Liljeberg P, Plosila J, Tenhunen H. LastZ: An Ultra Optimized 3D Networks-on-Chip Architecture. Proceedings of the 2011 14th Euromicro Conference on Digital System Design; 2011; Oulu, Finland. Piscataway, NJ: IEEE; 2011.

[120] Richter DC, Schuster SC, Huson DH. OSLay: optimal syntenic layout of unfinished assemblies. Bioinformatics. 2007;23(13):1573–9. 10.1093/bioinformatics/btm153 17463020

[121] Assefa S, Keane TM, Otto TD, Newbold C, Berriman M. ABACAS: algorithm-based automatic contiguation of assembled sequences. Bioinformatics. 2009;25(15):1968–9. 10.1093/bioinformatics/btp347 PubMed Central PMCID: PMCPMC2712343. 19497936 PMC2712343

[122] Rissman AI, Mau B, Biehl BS, Darling AE, Glasner JD, Perna NT. Reordering contigs of draft genomes using the Mauve aligner. Bioinformatics. 2009;25(16):2071–3. 10.1093/bioinformatics/btp356 PubMed Central PMCID: PMCPMC2723005. 19515959 PMC2723005

[123] Munoz A, Zheng C, Zhu Q, Albert VA, Rounsley S, Sankoff D. Scaffold filling, contig fusion and comparative gene order inference. BMC Bioinformatics. 2010;11(1):304 10.1186/1471-2105-11-304 20525342 PMC2902449

[124] Husemann P, Stoye J. r2cat: synteny plots and comparative assembly. Bioinformatics. 2010;26(4):570–1. 10.1093/bioinformatics/btp690 PubMed Central PMCID: PMCPMC2820676. 20015948 PMC2820676

[125] Lu CL, Chen K-T, Huang S-Y, Chiu H-T. CAR: contig assembly of prokaryotic draft genomes using rearrangements. BMC Bioinformatics. 2014;15:381 10.1186/s12859-014-0381-3 PubMed Central PMCID: PMCPMC4253983. 25431302 PMC4253983

[126] Bosi E, Donati B, Galardini M, Brunetti S, Sagot M-F, Lió P, et al MeDuSa: a multi-draft based scaffolder. Bioinformatics. 2015;31(15):2443–51. 10.1093/bioinformatics/btv171 25810435

[127] Kolmogorov M, Raney B, Paten B, Pham S. Ragout-a reference-assisted assembly tool for bacterial genomes. Bioinformatics. 2014;30(12):i302–9. 10.1093/bioinformatics/btu280 PubMed Central PMCID: PMCPMC4058940. 24931998 PMC4058940

[128] Zeng F, Yao L, Chen Z, Qi H. A Distributed and Shortest-Path-Based Algorithm for Maximum Cover Sets Problem in Wireless Sensor Networks. Proceedigns of the 10th International Conference on Trust, Security and Privacy in Computing and Communications; 2011; Changsha, China. Piscataway, NJ: IEEE; 2011.

[129] Alekseyev MA, Pevzner PA. Breakpoint graphs and ancestral genome reconstructions. Genome Res. 2009;19(5):943–57. 10.1101/gr.082784.108 PubMed Central PMCID: PMCPMC2675983. 19218533 PMC2675983

[130] Kolmogorov M, Armstrong J, Raney BJ, Streeter I, Dunn M, Yang F, et al Chromosome assembly of large and complex genomes using multiple references. Genome Res. 2018 10.1101/gr.236273.118 30341161 PMC6211643

[131] Bertoni A, Valentini G. Discovering multi–level structures in bio-molecular data through the Bernstein inequality. BMC Bioinformatics. 2008;9(Suppl 2):S4 10.1186/1471-2105-9-s2-s4 18387206 PMC2323667

[132] Kolmogorov V. Blossom V: a new implementation of a minimum cost perfect matching algorithm. Math Program Comput. 2009;1(1):43–67. 10.1007/s12532-009-0002-8

[133] Yuan Y, Bayer PE, Batley J, Edwards D. Improvements in Genomic Technologies: Application to Crop Genomics. Trends in Biotechnology. 2017;35(6):547–58. 10.1016/j.tibtech.2017.02.009 28284542

[134] Wachi N, Matsubayashi KW, Maeto K. Application of next-generation sequencing to the study of non-model insects. 2018;21(1):3–11. 10.1111/ens.12281

[135] Tello-Ruiz MK, Naithani S, Stein JC, Gupta P, Campbell M, Olson A, et al Gramene 2018: unifying comparative genomics and pathway resources for plant research. Nucleic Acids Res. 2018;46(D1):D1181–D9. Epub 2017/11/23. 10.1093/nar/gkx1111 29165610 PMC5753211

[136] Koren S, Rhie A, Walenz BP, Dilthey AT, Bickhart DM, Kingan SB, et al De novo assembly of haplotype-resolved genomes with trio binning. Nature Biotechnology. 2018;36:1174 10.1038/nbt.4277 30346939 PMC6476705

